# Dental Implant Failure Risk in Patients with Bruxism—A Systematic Review and Meta-Analysis of the Literature

**DOI:** 10.3390/dj13010011

**Published:** 2024-12-26

**Authors:** Josephine A. Ionfrida, Hanna L. Stiller, Peer W. Kämmerer, Christian Walter

**Affiliations:** 1 Department of Oral and Maxillofacial Surgery, University Medical Center Mainz, Augustusplatz 2, 55131 Mainz, Germanywalter@mainz-mkg.de (C.W.); 2 Oral and Maxillofacial Surgery, Mediplus Clinic, Haifa-Allee 20, 55128 Mainz, Germany

**Keywords:** bruxism, dental implants, implant failure, mechanical complications, systematic review, risk factor

## Abstract

**Background/Objectives:** Recent research has indicated that placing dental implants in patients diagnosed with bruxism has led to higher rates of implant failure. This study aimed to provide more accurate knowledge about the relationship between bruxism and implant loss in patients (number, age, gender) with different numbers of implants and prosthetic restorations, considering the follow-up time, compared to non-bruxers. **Methods:** A systematic search was conducted in PubMed and Cochrane Library using the keyword combination “dental implant”, “bruxism”, and “implant failure”. This search had no language or time restrictions. **Results:** The review included 15 papers, which were divided into four groups. The first group focused on studies regarding implant loss regardless of the superstructure. In the second group, research on implant-supported removable prostheses was examined. The third group consisted of a single study specifically addressing implant-supported single crowns. Lastly, the fourth group comprised two studies investigating implant-supported single crowns and fixed partial dentures. Only three out of the fifteen studies failed to find a correlation between bruxism and implant failure. The meta-analysis shows a significant pooled effect across the included studies, with an odds ratio of 4.68. **Conclusions:** Bruxism is a probable risk factor for mechanical issues in implant-supported prostheses and implant-supported crowns. Additionally, elevated failure rates have been documented in patients with bruxism.

## 1. Introduction

The term “bruxism” is derived from the Greek word “brygmós”, which translates to “gnashing of teeth” [1]. According to an international expert consensus from 2013, bruxism is defined as a “repetitive jaw-muscle activity characterized by clenching or grinding of the teeth and/or by bracing or thrusting of the mandible”. Bruxism can occur both during sleep and wakefulness. In 2018, the definition was expanded, clarifying the differences between awake bruxism (AB) and sleep bruxism (SB). AB is defined as “repeated or sustained tooth contact and/or bracing or thrusting of the mandible without tooth contact”, while sleep bruxism is defined as “rhythmic (phasic) or non-rhythmic (tonic) movement of the jaw muscles”. The terms “parafunction” and “movement disorder” are now considered outdated and are no longer preferred due to their negative associations. The phrase “bracing or thrusting of the mandible” is intended to include patients who wear prostheses or implant-supported dental restorations [2,3,4].

There are many theories about the etiology of bruxism. The assumption that peripheral factors such as malocclusion of the teeth or facial bone morphology significantly contribute to the development of bruxism is increasingly diminishing [4,5,6]. Risk factors for bruxism being discussed more frequently include stress, anxiety, sleep disorders, genetic factors, nicotine, alcohol, and drug consumption [4,5,7,8,9].

The prevalence of bruxism varies depending on the method of investigation. Therefore, literature reports for adults range from 12.8% ± 3.1% for SB and from 22.1% to 31% for AB. Different diagnostic procedures include history taking, clinical examination, or instrumental diagnostics using electromyography (EMG) or polysomnography (PSG) [10,11]. These procedures help estimate the likelihood of bruxism:

- Positive indications from self-reported history/questionnaires = possible SB/AB
- Positive indications from self-reported history/questionnaires + positive clinical indications = probable SB/AB
- Positive indications from self-reported history/questionnaires + positive clinical indications + positive instrumental findings (PSG, EMG, ideally with video or audio recordings) = definitive SB/AB [2,10,12].

Implants have become integral to today’s dental treatment spectrum and are now the method of choice for tooth replacement. Patients hope for unrestricted chewing function and optimum esthetics, which go hand in hand with a gain in quality of life. However, despite the excellent current data and often very good expertise of the dentist, the insertion of implants is often associated with complications. The literature on dental implant placement and maintenance in patients with bruxism is still limited. In most cases, bruxism is mentioned as one of many risk factors, with only a few studies exclusively investigating bruxism. This review aims to provide a systematic overview of the current data to address the long-term care of patients with bruxism and to answer the question whether there is a connection between bruxism and implant loss in patients (number, age, gender) with different numbers of implants and prosthetic restorations, considering the follow-up time, compared to non-bruxers.

## 2. Materials and Methods

This review aims to identify the literature that deals with the role of bruxism as a risk factor for dental implants. For this purpose, a PubMed and Cochrane Library search with the MeSh-Term combination “dental implant”, “bruxism”, and “implant failure” was conducted up to 1 November 2024. There was no period or language restriction. This study was previously registered on PROSPERO (ID: CRD42024617151). The manuscript was revised according to the AMSTAR 2 tool [13].

The search yielded 154 results, which JI systematically analyzed. The selected papers were subsequently reviewed again by the second author, HLS. If there were conflicting opinions, the articles were reviewed and discussed with CW regarding a final inclusion/exclusion decision. The inclusion and exclusion criteria were:

Inclusion criteria:

- Information on failure rates in bruxers/non-bruxers
- Sufficient information on the criteria used for the diagnosis of at least probable bruxism
- Randomized and non-randomized controlled studies
- Cohort studies
- Retrospective studies
- Case series with at least 10 patients
- Exclusion criteria:
- Reviews
- Case reports
- Animal studies
- In-vitro studies
- Guidelines and recommendation papers

### 2.1. Study Selection and Data Extraction

The literature was evaluated according to the PICO scheme of evidence-based medicine, which is broken down in this review as follows: P = patient/population (number, age, gender); I = intervention (number of implants, follow-up time, prosthetic restoration); C = comparison (bruxers vs. non-bruxers); and O = outcome (is there a connection between bruxism and implant loss?). The data extraction for the included studies was conducted by the authors. Specifically, the following data were extracted: study design, patient characteristics intervention (how many implants were placed), follow-up time, comparison of the investigated factors (implant failure and bruxism as a risk factor), conclusion/outcome of the study (is there a connection between implant failure and bruxism), and points of strength and weakness, including risk of bias of the included studies.

### 2.2. Risk-of-Bias Analysis

The risk-of-bias analysis of the included studies was conducted based on the Newcastle–Ottawa Scale. The NOS scale evaluates scientific cohort and case-control studies in three categories: selection, comparability, and outcome. The NOS scale contains eight items. For one item, the maximum score is one point (star). Only for the item of comparability is it possible to score 2 points. This leads to a maximum score of nine points (Table 1 and Table 2).

### 2.3. Meta-Analysis

A meta-analysis was conducted using an online tool (www.metaanalysisonline.com, accessed on 20 November 2024). This review was performed in accordance to the PRISMA (Preferred Reporting Items for Systematic Reviews and Meta-Analyses) guidelines.

## 3. Results

**Figure 1:**
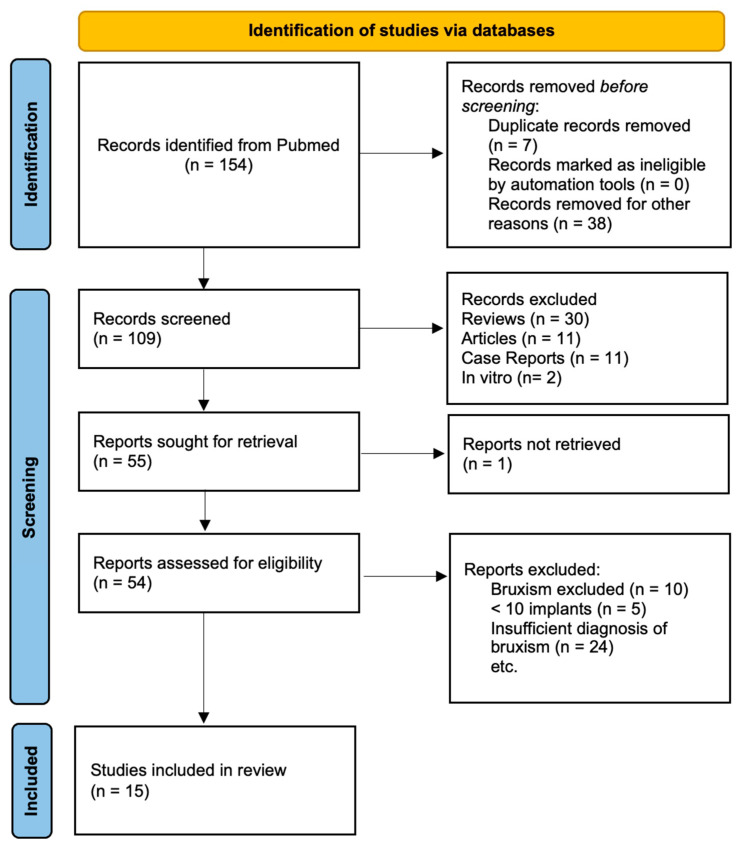
Literature selection process according to the PRISMA flow diagram.

The literature selection process can be found in Figure 1. Of the initial 154 search results, the abstracts were screened, whereupon 7 duplicates were removed, and 21 results were excluded because they were not related to the topic of bruxism along with a further 18 because the outcome of implant loss was not investigated (*n* = 38). A total of 30 results were reviews, 11 were case reports, 2 were in-vitro studies, and 11 were articles which consisted of authors’ opinions or statements only. Five search results had less than ten implants or bruxism was considered an exclusion criterion for the population (*n* = 10). Insufficient diagnosis of bruxism was made in 24 articles. In the end, 15 studies were identified that met all the above-mentioned criteria.

### 3.1. Descriptive Analysis

The included studies were categorized into four groups. In the first group (Table 3), studies are described that exclusively consider implant loss independently of the superstructure [14,15,16,17,18,19,20,28]; in the second group (Table 4), implant-supported removable prostheses were examined [21,22,23,24]; and the third group (Table 5) comprises one study on implant-supported single crowns only [25]. The fourth and last group shows two studies on implant-supported single crowns and fixed partial dentures [26,27] (Table 6).

#### 3.1.1. Studies on Bruxism as a Factor of Implant Loss

In the first group, a total of more than 27,803 implants were included, which were placed in 8692 patients (Table 3). The smallest study describes 227 implants in 98 patients [17] and the largest study 10,099 implants in 2670 patients [16]. In one study, the number of implants was not described [19]. The mean follow-up time across all studies ranged from 3 to 16 years [17]. All the studies were published between 2016 and 2018.

While four of the eight studies explicitly investigated the relationship between bruxism and implant failure [14,19,20,28], one study examined several factors generally associated with overload (unfavorable occlusion due to premature occlusal contact, bruxism, etc.) [15]. Another study investigated the cluster behavior of dental implant failures, where a patient was considered to have a cluster failure if they had at least three dental implant failures [18]. The other two studies investigated general risk factors such as implant diameter, length, bruxism, and abutment angulation [16,17]. Seven of the eight studies suggested a correlation between bruxism and implant failure. In one study, the diagnosis of bruxism was based on self-assessment [14]; in all other studies, clinical examinations or photo documentation was used [19]. In the works of Chrcanovic et al., identical patient cohorts were examined for different research questions [16,18,20,28]. This could represent a multiple publication bias.

#### 3.1.2. Studies on Bruxism as a Factor of Implant Loss and Prothesis Failure

The second group consists of studies that investigated implant failure and prosthesis failure (Table 4). A total of 7202 implants were placed in 1524 patients, supporting 2399 prostheses. In one study, the number of implants placed was not described [24]. The largest population studied contained 709 [22], and the smallest 85 patients [21]. The follow-up time varied and ranged from 0.2 months up to 26 years [21]. The three studies by Chrcanovic et al. focus on the relationship between bruxism and implant or prosthesis failure, and the same patient cohort is examined from different perspectives [21,22,23]. The last study focused on bruxism and mechanical complications (loosening/fracture of artificial teeth or screw loosening) [24]. A clinical diagnosis of bruxism was made in three of the four studies [22,23]. In one of them, the bilateral maximum occlusal force was measured using a cross-arch compressive force transducer [24]. The last study was based on self-reported bruxism [21]. In three out of four studies, a positive correlation between bruxism and implant and/or prosthesis failure was found [21,22,23]. The study analyzing mechanical complications did not reveal an association with bruxism [24].

#### 3.1.3. Studies on Bruxism as a Factor of Implant Loss in Implant-Supported Single Crowns

The third group contains only one study, in which 358 implant-supported single crowns were observed in 278 patients with a mean follow-up time of 56.5 ± 29.7 months [25] (Table 5). The study focused on the relationship between bruxism and implant failure or technical complications such as prosthetic abutment fracture, crown decementation, or prosthetic screw fracture. Bruxism diagnosis was based on self-reports. In this study, bruxism was associated with a higher risk of failure of implant-supported single crowns, with a hazard ratio of 2.962.

#### 3.1.4. Studies on Bruxism as a Factor of Implant Loss in Implant-Supported Single Crowns and (FPD) Fixed Partial Dentures

The fourth and last group contains two retrospective studies, which dealt with the effect of bruxism on implant-supported crowns and fixed partial dentures (Table 6). A total of 545 implants were analyzed. While the first study [26] found a significant effect of bruxism on the occurrence of complications, no correlation was found in the second study [27].

### 3.2. Qualitative Analysis

As the included studies are not randomized, a qualitative assessment of the clinical work using the Newcastle–Ottawa Scale, specifically designed for this purpose, seems appropriate. The University of Ottawa developed this scale in collaboration with the University of Newcastle to evaluate study design, content, and usability [29]. Although the scale is primarily intended for cohort and case-control studies, other observational studies, such as cross-sectional studies, are also classified under these subgroups and assessed accordingly. The evaluation distinguishes between three main categories: Selection, Comparability, and Exposure/Outcome. Each category includes approximately three to four questions the reviewer must answer and assess for each study individually. Only in the Comparability category can two stars be awarded. The total number of stars results in a ranking of the studies (Table 1 and Table 2).

**Figure 2:**
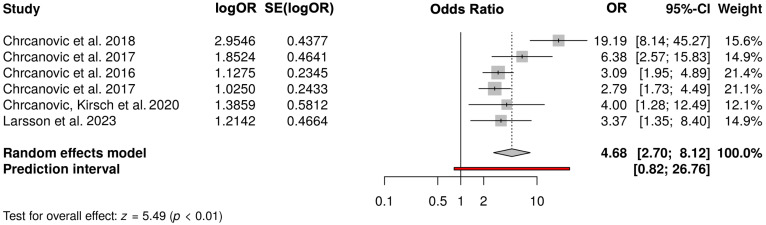
Meta-analysis; [16,18,20,21,25,28].

A meta-analysis would be attempted if studies with comparable comparisons reported the same outcome measures. For this review, six studies were included that compare the risk of implant loss in bruxers versus non-bruxers (Figure 2). In studies where the odds ratio was not provided, it was calculated by the reviewer based on the available data. The analysis included the Odds ratio with 95% confidence intervals, the logarithmic OR and its standard deviation (SE), and the weight of the respective studies. The pooled odds ratio in the random effects model is 4.68, with a 95% confidence interval of [2.70; 8.12].

## 4. Discussion

This review aimed to provide an overview of the literature on bruxism as a risk factor for implantation. To date, the literature on implantations in patients with bruxism remains limited. Bruxism is often mentioned as one of many risk factors, with only a few studies exclusively focusing on bruxism. Unfortunately, only retrospective studies address this topic. The results of the reviewed studies suggest that bruxism is a risk factor for both implant loss and prosthesis failure. Three of the fifteen studies found no correlation, but these three studies included aspects that could have influenced this outcome. In the study by Chatzopoulus et al., a large population (n = 2127) was examined [14], but the diagnosis of bruxism was solely based on self-reports. In the study by Coltro et al. [24], bruxism was clinically diagnosed, but it involved only a small population (n = 88). In the study by Tawil et al., parafunction was evaluated based on the patient’s awareness of any bruxing habit and signs of occlusal trauma (wear facets, temporomandibular joint disorders). Patients were classified as non-bruxers, occasional bruxers, or heavy bruxers [27]. However, the criteria for categorizing patients into the respective groups remain unclear, and the group sizes also differ greatly (22.6% bruxer, 5.9% occasional bruxer group, 71.4% non-bruxer).

The pooled odds ratio in the random effects model is 4.68, with a 95% confidence interval of [2.70; 8.12]. This indicates that the effect is statistically significant. The Prediction Interval is [0.82; 26.76]. This gives the range within which the effect in a future study is expected to fall with 95% probability. The meta-analysis shows that the pooled effect across the included studies is significant, with an odds ratio of 4.68. This means that the event under study (based on the research question) is approximately 4.68 times more likely to occur in the exposed groups compared to the control groups.

The Newcastle–Ottawa Scale (NOS) is often used in systematic reviews and meta-analyses to assess the quality of the included studies. Studies with a higher number of stars are considered high-quality. In this review, all studies scored between seven and nine points, indicating the high quality of the publications. Although the NOS is a useful tool for quality assessment, there are criticisms, such as the subjectivity in the evaluation and the fact that the scale does not always capture the diversity and complexity of epidemiological studies. Additionally, not all types of bias (e.g., publication bias) can be detected with the NOS.

Overall, diagnosing bruxism in clinical practice poses a challenge. Instrumental diagnostics such as electromyography (EMG) or polysomnography (PSG) entail high technical, financial, and time costs for daily practice [4]. Therefore, diagnosis mainly relies on clinical examination by the practitioner or self-report.

It should be noted that most of the included studies in this review were published by Chrcanovic et al. It is reasonable to assume that the same patient cohorts were analyzed under different aspects, which makes the results susceptible to bias.

Overall, the findings of this review align with the existing reviews, such as the review by Manfredini et al. on bruxism as a risk factor for implants from 2014, including 21 studies [30]. The selection in this 2014 review was limited to studies exclusively addressing the influence of bruxism on implants. Seven of the described studies addressed technical complications. The total number of placed implants was 2590 in over 700 patients, followed up for at least four years. Four of these studies suggested a correlation between bruxism and mechanical complications after implantation, such as the study by Maló et al., where an odds ratio of 60.9 (CI: 21.4–173; *p* ≤ 0.0001) was found [31]. The other three studies found no correlation, with weaknesses including unclear criteria for diagnosing bruxism. The study by Maló et al. lacked a clear definition of bruxism diagnosis, which led to its exclusion from this review. Another review by Salvi et al., from 2014, investigated bruxism as one risk factor among ten other factors [32]. Out of initially 3568 papers, 35 were finally included in the analysis, with bruxism described in five of them. Two clinical studies showed statistically significantly higher rates of technical or mechanical complications (17.3% and 23%) and higher loss rates (60% and 39%) in patients with bruxism compared to those without. Two other publications showed a tendency towards more complications in bruxers, while one study found no correlation. Two studies by De Bouver et al. and Tawil et al. were also included in this review, while the rest of the studies were excluded due to a lack of definition of bruxism.

One of the latest reviews was published in 2022 by Youssef et al., aiming to gain more precise knowledge about the influence of bruxism on implants [33]. Sixteen out of initially 343 papers were analyzed and categorized into three groups (implant complications n = 10, prosthetic complications n = 3, and implant plus prosthetic complications n = 3). Prosthetic complications included ceramic fractures, abutment deformations, and decementation of supra-constructions. All the studies except two found a correlation between the mentioned complications and bruxism. The most recent review by Häggman-Henrikson et al., which elaborated on 27 studies presenting data on 2105 implants in probable bruxers and 10,264 implants in non-bruxers, also concluded that bruxers show an increased risk of implant failure [34].

To develop robust research, future studies should focus on a clear and evident diagnosis of bruxism, unified units of measurement, and an appropriate protocol. Future studies focusing on bruxism as a risk factor for implant loss would be desirable, as it often plays only a minor role in the current literature.

When comparing this study with the existing reviews, a small discrepancy can be recognized within the search results and ultimately included studies. One reason for this could be the definition of bruxism in the respective papers. This review only included studies that included a precise definition of bruxism and its diagnosis.

Limitations of this systematic review include publication bias, which can skew the overall findings, heterogeneity among studies, which can complicate the comparison, and differing levels of study quality. Furthermore, this review relies on a literature search of only one database, possibly limiting the number of results.

## 5. Conclusions

Most of the existing literature on this topic sees an association between bruxism and implant failure and mechanical complications. However, since the diagnosis of bruxism is still very inconsistent and often relies on self-reports from patients, obtaining reproducible results can be challenging. Although an improvement in the study situation by means of prospective studies would be desirable, the take-home message is clear: patients showing signs of bruxism require special attention in the dental practice, regardless of potential implant treatments.

## Figures and Tables

**Table 1:** Cohort studies assessed on the NOS. * = one point, ** = 2 points.

Study	Selection				Comparability	Exposure			∑
	Question				Question	Question			
	(1)	(2)	(3)	(4)	(1)	(1)	(2)	(3)	
Chatzopoulos, G.S. and Wolff, L.F. 2020 [14]	*	*	*	*	**	*			7
Stoichkov, B. and Kirov, D. 2018 [15]	*	*	*	*	*	*	*		8
Chrcanovic, B.R., et al. 2018 [16]	*	*	*	*	**	*	*		8
Papi, P., et al. 2017 [17]	*		*	*	*	*	*	*	7
Chrcanovic, B.R., et al. 2017 [18]	*	*	*	*	**	*	*		8
Yadav, K., et al. 2016 [19]	*	*	*	*	*	*	*		7
Chrcanovic, B.R., et al. 2016 [20]	*	*	*	*	**	*	*	*	9
Chrcanovic, Kisch, B.R., J. and Larsson, C. 2020 [21]	*	*	*	*	**	*	*	*	9
Chrcanovic, Kisch, B.R., J. and Larsson, C. 2020 [22]	*	*	*	*	**	*	*	*	9
Chrcanovic, Kisch, B.R., J. and Larsson, C. 2020 [23]	*	*	*	*	**	*	*	*	9
Coltro, M.P.L., et al., 2018 [24]	*	*	*	*	**	*	*	*	9
Larsson, A., Manuh, J. and Chrcanovic, B.R. 2023 [25]	*	*	*	*	**	*	*	*	9
De Boever, A., et al. 2006 [26]	*	*	*	*	*	*	*	*	8
Tawil, G., Aboujaoude, N. and Younan, R. 2006 [27]	*	*	*	*	*	*	*		7

**Table 2:** Case-control studies assessed on the NOS. * = one point, ** = 2 points.

Study	Selection				Comparability	Exposure			∑
	Question				Question	Question			
	(1)	(2)	(3)	(4)	(1)	(1)	(2)	(3)	
Chrcanovic, B.R., et al. 2017 [28]	*	*	*	*	**	*	*	*	9

**Table 3:** Studies on bruxism as a factor of implant loss. (F = female; M = male; * = adjusted estimate).

Study	Population	Intervention	Aim of the Study	Does Bruxism Contribute?	Conclusion	Points of Strength	Points of Weakness
Chatzopoulos et al. (2020) [14]	2127 patients (1055 M; 1072 F)	4519 implants (follow-up up to 76 months)	To retrospectively investigate the association between symptoms of temporomandibular disorder bruxism with the risk of implant failure	No	Bruxism does not contribute to implant failure *	Large sample size	Self-reported data on bruxism
Stoichkov et al. (2018) [15]	101 patients (55 M; 46 F)	218 implants (3- to 6-year follow-up)	To analyze the possible causative factors contributing to implant body fracture	Yes	Bruxism shows a statistically significant influence on implant fracture (*p* < 0.001)	Clinical diagnosis of bruxism	Risk factors not weighted
Chrcanovic et al. (2017) [16]	2670 patients	10099 implants (inserted between 1980 and 2014)	To determine the possible risk factors predisposing an implant to a higher fracture risk	Yes	Bruxers have a higher probability of presenting an implant fracture (OR 19.195) *	Multivariate analysis Bruxism diagnosis based on guideline	Age and sex distribution undefined Duplicate publication
Papi et al. (2017) [17]	98 patients (56 M; 33 F)	227 implants (10 to 16 years follow-up)	To assess the long-term survival rate of dental implants placed in patients presenting mechanical risk factors	Yes	Higher failure rates in patients with bruxism (HR 2.9) *	Clinical diagnosis of bruxism	Small sample size
Chrcanovic et al. (2017b) [18]	1406 patients (390 M; 376 F)	8337 implants (inserted between 1980 to 2014)	To analyze cluster behavior of dental implant failures among patients and to assess the possible risk factors influencing this phenomenon	Yes	Bruxism is a potential risk factor for cluster behavior (OR 6.376) *	Minimum of 3 implants per patientLarge sample size	Duplicate publication
Yadav et al. (2016) [19]	1100 patients (490 M; 610 F)	Unidentified number of implants inserted between 2004 to 2014	To assess the complications occurring in dental implants in patients with and without bruxism	Yes	Success of dental implant is significantly affected by bruxism (OR 2.45)	Patient at risk assessment Bruxism diagnosis based on clinical history and photographs	Number of implants unknown Implant failure not defined
Chrcanovic et al. (2016) [20]	994 patients (478 M; 516 F)	3549 implants (variable follow-up)	To investigate the association between sleep and/or awake bruxism and the risk of dental implant failure, and to describe and compare the group of bruxers with non-bruxers	Yes	Bruxism was a statistically significant risk factor for implant failure (HR 3.396) *	Clinical diagnosis of bruxism Large sample size	Risk factors not weighted Duplicate publication
Chrcanovic et al. (2016) [28]	196 patients (98 M; 96 F)	427 implants in bruxers and 427 implants in non-bruxers	To analyze the complications of dental implant treatment in a group of patients with bruxism in comparison with a matched group of non-bruxers	Yes	Bruxism may increase the implant failure rate (OR 2.71)	Clinical diagnosis of bruxism	Other possible risk factors Duplicate publication

**Table 4:** Studies on bruxism as a factor of implant loss and prosthesis failure. * = adjusted estimate.

Study	Population	Intervention	Aim of the Study	Does Bruxism Contribute?	Conclusion	Points of Strength	Points of Weakness
Chrcanovic et al. (2020) [21]	85 patients (41 M; 44 F)	164 implants (follow-up 7 months to 26 years)	To assess clinical outcomes of fixed dental prostheses (FDPs) with combined implant and tooth support in relation to implant-, site-, patient-, and prosthetic-risk factors (such as bruxism)	Yes	Bruxism was suggested to be the only factor to statistically significantly exert some influence on the occurrence of prosthesis failure (HR 2.890)	Large sample size	Risk factors not weighted Bruxism based on self-report Duplicate publication
Chrcanovic et al. (2020) [22]	709 patients (318 M, 391 F)	4797 implants 869 implant-supported full-arch fixed dental protheses (mean follow-up 10 y)	To assess the outcomes of full-arch fixed dental prostheses supported by dental implants	Yes	Patients with bruxism presented a statistically significant lower survival of implants than patients with no bruxism (*p* < 0.001, log-rank test)	Clinical diagnosis of bruxismLarge sample size Multiple variable assessment	Duplicate publication
Chrcanovic et al. (2020) [23]	642 patients (289 M; 353 F)	2241 implants 876 two to six units implant-supported fixed partial dentures (mean follow-up 9 y)	To assess the clinical outcomes of fixed partial dentures supported by dental implants	Yes	Bruxism was a factor exerting a higher risk of screw and implant fracture, and ceramic chipping *	Clinical diagnosis of bruxismLarge sample size Multiple variable assessment	Duplicate publication
Coltro et al. (2018) [24]	88 patients (29 M; 59 F)	94 screw-retained, metal-acrylic implant-supported fixed complete dentures (follow-up mean of 35.1 ± 18.3 month; range 0.2–67.9)	To measure the survival and success rates, identify risk factors for mechanical complications, and evaluate the patient’s perception of the impact of oral rehabilitation in patients with implant-supported fixed complete dentures (IFCD)	No	Mechanical complications were not associated with bruxism *	Bilateral maximum occlusal force measurement Clinical diagnosis of bruxism	Small sample size Short follow-up

**Table 5:** Studies on bruxism as a factor of implant loss in implant-supported single crowns. * = adjusted estimate.

Study	Population	Intervention	Aim of the Study	Does Bruxism Contribute?	Conclusion	Points of Strength	Points of Weakness
Larrson et al. (2023) [25]	278 patients	358 implant-supported single crowns (145 in males; 213 in females) (mean follow-up 56.5 ± 29.7 months)	To investigate the risk factors that could be associated with the occurrence of failure and technical complications of implant-supported single crowns	Yes	Bruxism was associated with a higher risk of failure of implant-supported single crowns (HR 2.962) *	Large sample size	Risk factors not weighted Bruxism based on self-report

**Table 6:** Studies on bruxism as a factor of implant loss in implant-supported single crowns and (FPD) fixed partial dentures. F = female; M = male).

Study	Population	Intervention	Aim of the Study	Does Bruxism Contribute?	Conclusion	Points of Strength	Points of Weakness
De Boever et al. (2006) [26]	105 patients (48 M; 57 F)	283 implants; 172 fixed reconstructions; 23 bruxers, 80 non-bruxers (in 2 patients bruxism was not determined)	To describe the prosthetic technical complications, the possible risk factors involved, and the type and costs of the interventions to repair the complications of fixed restorations on non-submerged ITI implants	Yes	More complications occurred in patients with bruxism (*p* < 0.001)	Clinical diagnosis of bruxism	Risk factors not weighted
Tawil et a. (2006) [27]	109 patients (44 M; 69 F)	262 implants supported 123 fixed restorations, 33 being single crowns (mean follow-up 53 months)	To determine the influence of crown-to- implant (C/I) ratio, dimension of the occlusal table, nature of opposing dentition, mesial and distal cantilever, mesiodistal dimension of the prostheses in relation to the number and distribution of the implants, veneering material, and parafunctional habits on the survival and complication rate of short machined-surface Brånemark implants	No	Although more complications occurred in the bruxer group, there was no statistical difference in the rate of complications in the different bruxism groups examined (*p* = 0.51)	Clinical diagnosis of bruxism Patients divided into three groups (nonbruxer 71.4%; occasional bruxers 5.9% heavy bruxers 22.6%)	Unclear categorization of the groups Uneven distribution of the number of patients in the groups
